# Association of parents’ and children’s physical activity and sedentary time in Year 4 (8–9) and change between Year 1 (5–6) and Year 4: a longitudinal study

**DOI:** 10.1186/s12966-017-0565-0

**Published:** 2017-08-17

**Authors:** Russell Jago, Emma Solomon-Moore, Corrie Macdonald-Wallis, Janice L. Thompson, Deborah A. Lawlor, Simon J. Sebire

**Affiliations:** 1 Centre for Exercise, Nutrition & Health Sciences, School for Policy Studies, University of Bristol, 8 Priory Road, Bristol, BS8 1TZ UK; 2 School of Sport, Exercise and Rehabilitation Sciences, University of Birmingham, Birmingham, B15 2TT UK; 3 MRC Integrative Epidemiology Unit at the University of Bristol, Oakfield House, Oakfield Grove, Bristol, BS8 2BN UK; 4 School of Social and Community Medicine, University of Bristol, Canynge Hall, Whiteladies Road, Bristol, BS8 2PS UK

**Keywords:** Physical activity, Children, Cohort, Parents, Sedentary behavior

## Abstract

**Background:**

Parents could be important influences on child physical activity and parents are often encouraged to be more active with their child. This paper examined the association between parent and child physical activity and sedentary time in a UK cohort of children assessed when the children were in Year 1 (5–6 years old) and in Year 4 (8–9 years old).

**Methods:**

One thousand two hundred twenty three children and parents provided data in Year 4 and of these 685 participated in Year 1. Children and parents wore an accelerometer for five days including a weekend. Mean minutes of sedentary time and moderate-to-vigorous intensity physical activity (MVPA) were derived. Multiple imputation was used to impute all missing data and create complete datasets. Linear regression models examined whether parent MVPA and sedentary time at Year 4 and at Year 1 predicted child MVPA and sedentary time at Year 4. Change in parent MVPA and sedentary time was used to predict change in child MVPA and sedentary time between Year 1 and Year 4.

**Results:**

Imputed data showed that at Year 4, female parent sedentary time was associated with child sedentary time (0.13, 95% CI = 0.00 to 0.27 mins/day), with a similar association for male parents (0.15, 95% CI = −0.02 to 0.32 mins/day). Female parent and child MVPA at Year 4 were associated (0.16, 95% CI = 0.08 to 0.23 mins/day) with a smaller association for male parents (0.08, 95% CI = −0.01 to 0.17 mins/day). There was little evidence that either male or female parent MVPA at Year 1 predicted child MVPA at Year 4 with similar associations for sedentary time. There was little evidence that change in parent MVPA or sedentary time predicted change in child MVPA or sedentary time respectively.

**Conclusions:**

Parents who were more physically active when their child was 8–9 years old had a child who was more active, but the magnitude of association was generally small. There was little evidence that parental activity from three years earlier predicted child activity at age 8–9, or that change in parent activity predicted change in child activity.

**Electronic supplementary material:**

The online version of this article (doi:10.1186/s12966-017-0565-0) contains supplementary material, which is available to authorized users.

## Background

Children who are physically active have lower levels of risk factors for cardio-metabolic disease, lower risk of obesity and improved psychological well-being [1]. The UK Chief Medical Officers have recommended that all children and adolescents should engage in at least 60 min of moderate-to-vigorous-intensity physical activity (MVPA) per day and reduce sedentary time [2]. Large national surveys from the UK [3] and USA [4, 5] indicate that many children do not engage in the recommended hour per day of MVPA [2] and that both boys and girls become less active as they get older [6]. Ensuring that children are active, stay active and limit sedentary time has, therefore, been recognized as a public health priority [6]. Recent systematic reviews and meta-analyses indicate that interventions to increase physical activity and reduce sedentary time among children and adolescents have demonstrated limited efficacy [7, 8]. The reviews conclude that there is still much to be learned about the origins of children’s physical activity, how it could be changed and that new, improved behavior change programs are needed.

Parents are often blamed for the inactivity of their children [9, 10], with the media calling for parents to spend more time being active with their children. These statements can be counter-productive, leading to some parents feeling helpless as they may have insufficient time, resources and/or knowledge of how to help their children to be active [11–13]. Parent-child activity is, however, often promoted. For example, Sport England are currently investing £40 million in projects that promote physical activity for children with their parents [14]. The potential utility of these schemes and particularly whether promoting physical activity for parents and children together is likely to be effective is unclear.

Several studies have reported associations between parent and child physical activity [15–21]. These associations have been interpreted as evidence of parents and children being active together and used to advocate for parent-child physical activity interventions [20]. The bulk of the studies have, however, either used self-report methods, small samples or been conducted with pre-school aged children in cross-sectional study designs [20, 21]. Studies that have included older children have generally reported comparatively low associations between parent and child accelerometer-derived estimates of physical activity [15–19]. For example, correlations between parents’ and children’s MVPA were generally low (i.e., *r* < 0.08) [20, 22], and in our previous analyses we reported that every 10 min of parental MVPA was associated with just one additional minute of child MVPA [16]. Most studies have focused on the start or end of primary (elementary) school, resulting in a paucity of information on how parent activity during the middle primary school years is associated with child activity. This gap is particularly important as children’s physical activity levels progressively decline during primary school [6, 23, 24] and strategies are needed to stop this decline before the transition to secondary school [25]. Furthermore, there is absence of prospective data.

In this paper, we examined the association between objectively-assessed MVPA and sedentary time of Year 4 (8–9 year old) children and their parents. We also sought to determine whether parental MVPA and sedentary time during Year 1 (5–6 years old) predicted child MVPA and sedentary time at Year 4, and if change in parental behavior was associated with change in child behavior. Finally, we examined if there were any differences in associations for male and female parents, which may suggest a need to tailor behavior change interventions to parental gender.

## Methods

The current analyses used data from the B-PROACT1V study [16, 17, 26, 27]. The study examined the physical activity behaviors of children and their parents as the children progressed through primary school. Between 2012 and 2013, data were collected from 1299 children from 57 schools in the greater Bristol (UK) area who were in Year 1 (5–6 years of age). Between March 2015 and July 2016, data were collected from 1223 children in 47 of the original schools. The study received ethical approval from the School for Policy Studies Ethics Committee at the University of Bristol and written parent consent was received for all participants [28].

### Parent and child accelerometer measures

Children and at least one of their parents wore a waist-worn ActiGraph wGT3X-BT accelerometer for five days, including two weekend days, in Year 1 and then again in Year 4. Accelerometer data were processed using Kinesoft (v3.3.75; Kinesoft, Saskatchewan, Canada) in 60-s epochs. To enable comparison with international datasets [6], for inclusion in analysis, at least three valid days of data must have been provided, where a valid day was defined as at least 500 min of data, after excluding intervals of ≥60 min of zero counts allowing up to two minutes of interruptions. The average number of sedentary and MVPA minutes per day were derived using the Evenson population-specific cut points for children (≥2296 cpm) [29], and the Troiano cut points (≥2020 cpm) for adults [30].

### Parent and child characteristics

Child height was measured to the nearest 0.1 cm using a SECA Leicester stadiometer (HAB International, Northampton) and weight was measured to the nearest 0.1 kg using a SECA 899 digital scale (HAB International, Northampton). These were used to derive the child’s body mass index (BMI) as weight (kg)/ height (m)^2^, and this was converted to an age- and gender-specific standard deviation score [31]. Parents completed a questionnaire, which included information on the child’s gender, date of birth, number of siblings and the parent’s date of birth, height and weight. Where child’s date of birth was missing (21% of all children), they were assigned the median age of 6.0 years at Year 1, and 9.0 years at Year 4. Indices of Multiple Deprivation (IMD) scores, based upon the English Indices of Deprivation (http://data.gov.uk/dataset/index-of-multiple-deprivation), were assigned to each family based on their reported home postcode, where higher IMD scores indicate a greater level of deprivation.

### Statistical analysis

A description of the study design and reasons for incomplete data at the two timepoints has been reported previously [23, 32]. Briefly, however, there was considerable pupil movement between schools between Year 1 and 4 and different families consenting to participate in the two different waves. To account for missing data two separate multiple imputation models were used, with the first including the 1223 children who participated in the study in Year 4 (but not necessarily in Year 1) and the second including the 685 children who participated in the study in both Year 1 and Year 4. The first imputation was used to examine the association between parent and child physical activity in Year 4. This included relevant parental exposures (female and male parent sedentary and MVPA minutes per day), child outcomes (sedentary and MVPA minutes per day) and co-variables measured at Year 4 (child age, BMI z score, IMD, and female and male parent age and BMI).

The second imputation was used to examine the association between parent physical activity in Year 1 and child physical activity in Year 4, and change in child physical activity from Year 1 to Year 4. The imputation model therefore included child and female and male parent sedentary and MVPA minutes per day at both Year 1 and Year 4, as well as child age, BMI z-score, IMD, female and male parent age and BMI at Year 1 and Year 4. Changes in child and parent sedentary time and MVPA between Year 1 and Year 4 were imputed passively from their values at Year 1 and Year 4.

As there is consistent evidence that physical activity patterns differ by gender [5, 33–35] both sets of imputations were run separately for boys and girls to allow for associations to differ by child gender and included a school indicator variable to account for clustering within schools. In both cases, we created 20 imputed datasets using 20 cycles of regression switching and combined regression coefficients across the imputed datasets using Rubin’s rules [36].

We used linear regression models to examine the associations of interest, with robust standard errors to account for clustering within schools. We fitted models for boys and girls combined, as well as separately by gender and used compared point estimates and their 95% confidence intervals between girls and boys, as well as computing a Wald test to assess evidence of interaction by gender. In Model 1 we adjusted only for the child’s gender and age. Model 2 was additionally adjusted for the child’s BMI z-score, household IMD score, number of siblings and the parent’s age and BMI. The covariables measured at Year 4 were used for the models in which parent’s physical activity at Year 4 was the exposure. Covariables measured at Year 1 were used for models which analyzed parent’s physical activity at Year 1, or change in parent’s physical activity between Year 1 and Year 4, as the exposure.

Regression analyses were repeated, restricting to children and parents who had complete data for all exposures, outcomes and covariables, and compared with the multiple imputation analysis. All analyses were performed in Stata version 14.0 (StataCorp, 2015).

## Results

The characteristics of all children and parents who participated in Year 4 and those who participated in both Year 1 and Year 4 in the observed and multiple imputation datasets are shown in Table 1. The distributions of characteristics measured at Year 4 were comparable in the full set of all 1223 children who took part at Year 4 and the 685 who also took part in Year 1. Generally, the distributions of characteristics in the multiple imputation data were very similar to those in the observed data, with the exception of the change in male parents’ sedentary and MVPA minutes per day between Year 1 and Year 4, for which the means differed and standard deviations were much higher in the multiple imputation data compared with the observed data.

**Table 1:**
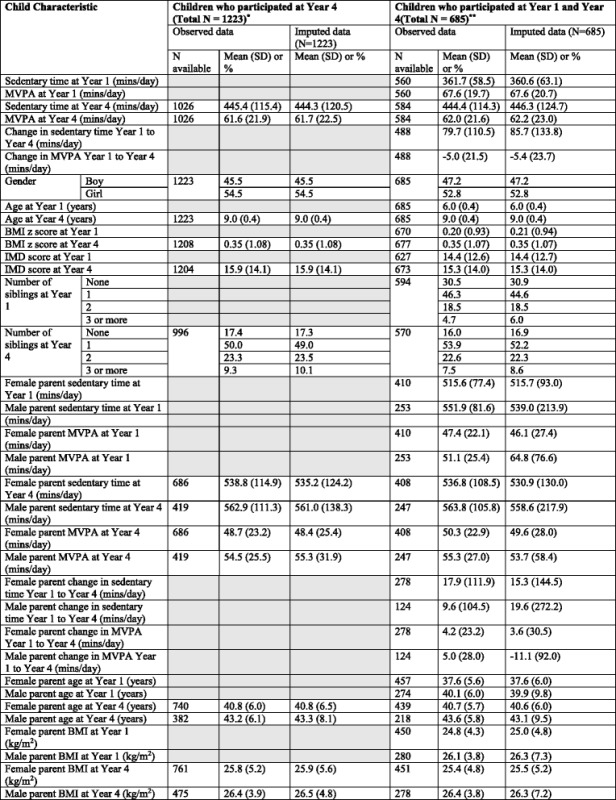
Characteristics of the children and their parents in observed and multiple imputation datasets

^*^Used for analysis of parent’s physical activity in Year 4 and children’s physical activity in Year 4. Characteristics not included in this analysis are shaded out in this column

^**^Used for analysis of parent’s physical activity in Year 1 and children’s physical activity in Year 4, as well as change in parent’s physical activity and change in children’s physical activity between Year 1 and Year 4

Table 2 shows the associations of parents’ sedentary time at Year 1 and Year 4 with the child’s sedentary time in Year 4 in the multiple imputation data. Female parents’ sedentary time at Year 4 was positively associated with children’s sedentary time at Year 4, in unadjusted and adjusted models. A similar-sized association was seen between the female parents’ sedentary time at Year 1 and the children’s sedentary time at Year 4, although the confidence intervals were wider and evidence weaker due to the smaller sample size for this analysis. These associations did not notably differ by child gender. Each additional minute per day of female parents’ sedentary time at Year 4 was associated with around an 8 s increase (95% CI: 0 to 16 s) in children’s sedentary time at Year 4, and each additional minute of female parents’ sedentary time at Year 1 was associated with a 10 s increase in children’s sedentary time (95% CI: -1 to 20 s). Male parents’ sedentary time at Year 4 was positively associated with boys’ sedentary time but not with girls’ sedentary time at Year 4, and there was statistical evidence to support this gender interaction. Each additional minute per day of male parents’ sedentary time associated with an extra 25 s of sedentary time per day in sons at Year 4 (95% CI: 10 to 39 s), but only 1 s of sedentary time in daughters (95% CI: -13 to 16 s). However, there was no evidence of an association between male parents’ sedentary time at Year 1 and the child’s sedentary time in Year 4 in boys or girls. Associations were similar when restricting to parent and child dyads with complete data (Additional file 1: Table S1).

**Table 2:** Mean difference (95% confidence interval) in the children’s average sedentary minutes per day in Year 4 associated with parents’ sedentary time in Year 4 and Year 1 using multiple imputation data^a^

Exposure		Child’s sedentary time in Year 4 (mins/day)			
		All Mean difference (95% CI)	Boys Mean difference (95% CI)	Girls Mean difference (95% CI)	*P* for gender interaction
Parent’s sedentary time in Year 4 (mins/day)		*N* = 1223	*N* = 556	*N* = 667	
Female parent	Model 1	0.13 (0.01, 0.26)	0.10 (−0.05, 0.25)	0.16 (−0.04, 0.36)	0.63
	Model 2	0.13 (0.00, 0.27)	0.10 (−0.06, 0.26)	0.17 (−0.03, 0.37)	0.63
Male parent	Model 1	0.15 (−0.02, 0.33)	0.40 (0.17, 0.64)	0.01 (−0.21, 0.23)	0.02
	Model 2	0.15 (−0.02, 0.32)	0.41 (0.18, 0.65)	0.02 (−0.22, 0.26)	0.03
Parent’s sedentary time in Year 1 (mins/day)		*N* = 685	*N* = 323	*N* = 362	
Female parent	Model 1	0.16 (−0.02, 0.34)	0.20 (−0.08, 0.48)	0.12 (−0.19, 0.43)	0.74
	Model 2	0.17 (−0.01, 0.34)	0.21 (−0.08, 0.50)	0.14 (−0.14, 0.42)	0.70
Male parent	Model 1	0.01 (−0.11, 0.12)	0.03 (−0.11, 0.17)	−0.04 (−0.30, 0.22)	0.60
	Model 2	0.01 (−0.12, 0.15)	0.04 (−0.15, 0.23)	−0.04 (−0.30, 0.22)	0.55

^a^Model 1 is adjusted for child’s age at Year 4 and gender; Model 2 is additionally adjusted for the child’s BMI z score, number of siblings, household IMD score, and the female/male parent’s age and BMI at Year 4 for models with parent’s sedentary time in Year 4 as the exposure, or for child’s BMI z score, number of siblings, household IMD score and the female/male parent’s age and BMI at Year 1 for models with the parent’s sedentary time in Year 1 as the exposure

The associations for parent and child MVPA in the multiple imputation data are shown in Table 3. Female parent MVPA at Year 4 was strongly positively associated with child MVPA at Year 4 in unadjusted and adjusted models, with similar-sized small associations in both boys and girls. However, female parent MVPA at Year 1 was not associated with child MVPA at Year 4. There was weak evidence that male parent MVPA at Year 4 was also associated with child MVPA at Year 4 (similarly in boys and girls) in Models 1 and 2, but male parent MVPA at Year 1 was not. Each additional minute per day of female parents’ MVPA at Year 4 was associated with around a 10 s increase in child MVPA (95% CI: 5 to 14 s), while an extra minute of male parent’s MVPA at Year 4 was associated with a 5 s increase in their child’s MVPA at Year 4 (95% CI: -1 to 10 s). Findings when restricting to those with complete data were generally similar, except that there was stronger evidence of a positive association between male parent MVPA at Year 4 and child MVPA at Year 4, and evidence of a positive association between female parent MVPA at Year 1 and girls’ MVPA at Year 4 but not boys’ (Additional file 1: Table S2).

**Table 3:** Mean difference in child Year 4 MVPA associated with parents’ MVPA in Year 4 and Year 1 using multiple imputation data^a^

Exposure		Child’s MVPA in Year 4 (mins/day)			
		All Mean difference (95% CI)	Boys Mean difference (95% CI)	Girls Mean difference (95% CI)	*P* for gender interaction
Parent’s MVPA in Year 4 (mins/day)		*N* = 1223	*N* = 556	*N* = 667	
Female parent	Model 1	0.16 (0.08, 0.24)	0.15 (0.02, 0.29)	0.16 (0.08, 0.25)	0.90
	Model 2	0.16 (0.08, 0.23)	0.16 (0.03, 0.28)	0.16 (0.07, 0.25)	0.94
Male parent	Model 1	0.08 (−0.01, 0.16)	0.06 (−0.10, 0.21)	0.10 (0.01, 0.18)	0.67
	Model 2	0.08 (−0.01, 0.17)	0.06 (−0.11, 0.23)	0.10 (0.00, 0.19)	0.57
Parent’s MVPA in Year 1 (mins/day)		*N* = 685	*N* = 323	*N* = 362	
Female parent	Model 1	0.04 (−0.04, 0.12)	−0.04 (−0.22, 0.14)	0.09 (−0.01, 0.18)	0.22
	Model 2	0.04 (−0.05, 0.12)	−0.03 (−0.22, 0.16)	0.08 (−0.02, 0.17)	0.32
Male parent	Model 1	−0.01 (−0.06, 0.03)	−0.03 (−0.10, 0.04)	0.06 (−0.00, 0.12)	0.05
	Model 2	−0.01 (−0.05, 0.04)	−0.03 (−0.10, 0.04)	0.06 (−0.01, 0.13)	0.07

^a^Model 1 is adjusted for child’s age at Year 4 and gender; Model 2 is additionally adjusted for the child’s BMI z score, number of siblings, household IMD score and the female/male parent’s age and BMI at Year 4 for models with parent’s sedentary time in Year 4 as the exposure, or for child’s BMI z score, number of siblings, household IMD score and the female/male parent’s age and BMI at Year 1 for models with the parent’s sedentary time in Year 1 as the exposure

The associations of change in parents’ sedentary time and parents’ MVPA between Year 1 and Year 4 with the child’s change in sedentary time and MVPA between Year 1 and Year 4 in the multiple imputation data are shown in Tables 4 and 5, respectively. There was no evidence in any models that change in either the female or male parents’ sedentary time or MVPA was associated with change in the child’s sedentary time or MVPA. Findings in those with complete data were generally similar (Additional file 1: Table S3 and Table S4), except that there was a positive association between the female parent’s MVPA change from Year 1 to Year 4 and the child’s change in MVPA between Year 1 and Year 4.

**Table 4:** Mean difference in the children’s change in sedentary minutes per day between Year 1 and Year 4 associated with parents’ change in sedentary time between Year 1 and Year 4 using multiple imputation (*N* = 685)^a^

Exposure		Child’s change in sedentary time Year 1 to Year 4 (mins/day)			
		All Mean difference (95% CI)	Boys Mean difference (95% CI)	Girls Mean difference (95% CI)	*P* for gender interaction
Parent’s change in sedentary time Year 1 to Year 4 (mins/day)		*N* = 685	*N* = 323	*N* = 362	
Female parent	Model 1	0.03 (−0.14, 0.20)	−0.03 (−0.25, 0.18)	0.11 (−0.17, 0.40)	0.40
	Model 2	0.03 (−0.14, 0.20)	−0.05 (−0.26, 0.17)	0.12 (−0.14, 0.39)	0.32
Male parent	Model 1	0.01 (−0.11, 0.12)	0.06 (−0.13, 0.25)	−0.05 (−0.26, 0.16)	0.35
	Model 2	0.00 (−0.12, 0.12)	0.07 (−0.12, 0.26)	−0.06 (−0.26, 0.15)	0.30

^a^Model 1 is adjusted for child’s age at Year 1 and gender; Model 2 is additionally adjusted for the child’s BMI z score, number of siblings, household IMD score and the female/male parent’s age and BMI at Year 1

**Table 5:** Mean difference (95% confidence interval) in the children’s change in moderate-to-vigorous physical activity minutes per day between Year 1 and Year 4 associated with parents’ change in moderate-to-vigorous physical activity between Year 1 and Year 4 using multiple imputation (*N* = 685)^a^

Exposure		Child’s change in MVPA Year 1 to Year 4 (mins/day)			
		All Mean difference (95% CI)	Boys Mean difference (95% CI)	Girls Mean difference (95% CI)	*P* for gender interaction
Parent’s change in MVPA Year 1 to Year 4 (mins/day)		*N* = 685	*N* = 323	*N* = 362	
Female parent	Model 1	0.06 (−0.04, 0.16)	0.14 (−0.03, 0.30)	0.01 (−0.13, 0.15)	0.22
	Model 2	0.06 (−0.05, 0.17)	0.14 (−0.03, 0.31)	0.01 (−0.13, 0.16)	0.28
Male parent	Model 1	0.02 (−0.03, 0.06)	0.02 (−0.03, 0.08)	0.00 (−0.10, 0.11)	0.73
	Model 2	0.02 (−0.03, 0.07)	0.02 (−0.04, 0.08)	0.00 (−0.12, 0.12)	0.73

^a^Model 1 is adjusted for child’s age at Year 1 and gender; Model 2 is additionally adjusted for the child’s BMI z score, number of siblings, household IMD score and the female/male parent’s age and BMI at Year 1

## Discussion

The findings in this paper demonstrate that there was a small association between the physical activity of parents and their Year 4 (8–9 years of age) child. Each minute of female parent MVPA was associated with an extra 10 s of child MVPA, while an extra minute of male parent MVPA was associated with only 5 extra seconds of child MVPA per day. In other words, every 10 min of female parent MVPA was associated with 1 min of child MVPA, while every 10 min of male parent MVPA was associated with 30 s. Conversely, female parents who were more sedentary at this time had children who were more sedentary, regardless of child gender, while male parents who were more sedentary specifically had more sedentary sons. These cross-sectional associations, were not replicated in longitudinal analyses. Parents who had been more physically active three years earlier, when their child was in Year 1, did not have a more active child in Year 4, and changes in parents’ physical activity and sedentary time did not correlate with changes in children’s behaviors over the three years. There was only weak evidence that female parents who were more sedentary three years earlier had children who were more sedentary in Year 4. Taken together these data challenge the notion that parents’ engagement with physical activity is an important determinant of their child’s activity levels.

In this study, there was little evidence that physical activity levels correlated more strongly in parent-child pairings of the same gender (i.e., that associations of the female parent’s physical activity with that of their child was stronger in girls than in boys, or that associations of male parent’s physical activity with that of his child were stronger in boys). The one exception was for male parent’s sedentary time when the child was in Year 4, where an extra minute of the parent sedentary time was associated with an extra 25 s of boy’s sedentary time but with little difference in daughter’s sedentary time.

The data presented in this study for Year 4 children (8–9 years old) are broadly similar to previous cross-sectional studies, which have reported correlations of around 0.1 between parents’ physical activity and the physical activity patterns of pre-school and young primary school age children [20, 22, 26]. Collectively, these findings suggest that there are very small associations between the physical activity and sedentary time of parents and children which may be a product of shared behavior such as walking to school or shared sedentary time during meals or homework, but overall the magnitude of associations is weak. As the mean minutes of parental MVPA was 48 min for mothers and 55 min for fathers, a 10% increase in mothers’ MVPA (~ 5 min) would approximately yield 50 additional seconds of child MVPA if the associations were maintained. Similarly, a 10% increase in fathers’ MVPA (5.5 min) would yield approximately 28 s of child MVPA. Thus, while there is strong evidence against the null hypothesis for these associations, the magnitude of association is very small and suggests that targeting parent activity to increase the child’s activity at Year 4 is unlikely to yield any potential health benefit at either the individual or population level. It is important to recognize that other forms of parental influence, such as providing logistic support for physical activity by enrolling children in activity programs and creating activity opportunities for children, have consistently been associated with a larger magnitude of increased physical activity among both boys and girls [12, 37–42]. Findings therefore suggest, simple strategies that focus on encouraging parents to be active at the same time, together with their child are unlikely to be sufficient to increase child physical activity [43]. More sophisticated strategies that take account of the key variables that influence both parent and child physical activity are likely to be required to change both behaviors.

The data presented in this paper suggest that there is no evidence of long-term association between the physical activity or sedentary time of children and their parents, and that change in parent behavior is not associated with change in child behavior. The lack of association could be because children and parents do not spend large amounts of time active together, with one GPS study reporting that parents and children spend only 2.4 min per day doing activity at the same time [18]. For example, parents may get the majority of their activity from walking and commuting while child activity may occur separately at school, in sport groups or more general active play [43–47]. The time that parents spend together may be very good for their relationship but it is likely to get greatly diluted by a range of other activities that they do separately. These findings do not downplay the potential importance of parent-child activity time as a source of fun, bonding, learning about rules and social development but may suggest that is not a big contributor to overall activity from a health perspective.

The evidence presented in this paper highlight a need to study the broader ways in which parents may influence their children’s physical activity. Potential mechanisms could be parenting practices (what a parent does), parenting styles (how messages are delivered), as well as a wide range of environmental factors such as access to green space, and psychosocial factors such as positive reinforcement and modelling of active behaviors. This wide range of variables may not be captured by individual theories of behavior change and are likely to require the development of more nuanced, parent-based models of physical activity promotion. The Family Ecological Model is one such model that has been applied to obesity prevention [48] and holds promise as a potential framework which could be adapted to focus specifically on understanding the ways in which parents influence child physical activity. As such, for the field to progress there is a need for the key elements of the framework, for which there is sufficient evidence, and the key evidence gaps, for which more empirical work is required, to either support or refute each variable’s role as a potential key predictor of child physical activity. In addition, there is also a need to develop new analytical frameworks for the assessment of these complex interactions which may not be immediately amenable for assessments via current methods. For example, it has recently been suggested that the lack of success of individual-focused interventions (such as physical activity) could be due to the failure to take account of the broader systems-level influences on behavior, and the ability of the system to adapt to interventions, thereby mitigating any effect that might be identified by current methodologies [49, 50]. This more complex and theoretically challenging work is likely to be needed to understand the very sophisticated and multi-layered human interactions between parents and their children which support or undermine physical activity.

### Strengths and limitations

The major strength of this study is the objectively-assessed physical activity data for children and their parents at two time points (Year 1 and Year 4). This has facilitated an examination of how parental MVPA and sedentary behavior at Year 1 is associated with child behavior at Year 4, as well as advancing the cross-sectional information by providing new information on Year 4 children. The study is however, limited by the provision of data from a single UK city area. We are unable to state that the data are representative of this region as we do not have data from non-responding schools, which limits our ability to generalize to other countries and contexts. As with all longitudinal studies, a proportion of the data were missing and this was higher for analyses involving both time points of data collection. We used multiple imputation to increase precision and potentially reduce bias in our estimates compared with analysis restricting to individuals with complete data. This assumes that data are missing at random, i.e., that any reasons for missingness can be explained by observed data [51]. It is not possible to test this assumption, but have included all exposures, outcomes, covariables and any variables that are predictive of missingness in our imputation models in order to increase the plausibility that it is correct. Finally, we used a hip worn accelerometer to identify sedentary time. There is currently a debate within the field [52] as to whether more nuanced definitions of forms of sedentary behavior are required, but as specific forms of behavior cannot be detected by accelerometer, further partitioning of the data into forms of sedentary behavior was not possible in this study.

## Conclusions

Our results challenge the notion that parental activity levels will influence their child’s physical activity and sedentary time, and suggest that interventions that aim to increase children’s activity levels by increasing their parent’s levels are unlikely to have marked impact on improving population levels of childhood activity.

## Additional file

Additional file 1:
**Table S1.** Mean difference (95% confidence interval) in the children’s average sedentary minutes per day in Year 4 associated with parents’ sedentary time in Year 4 and Year 1 for those with complete data. **Table S2.** Mean difference (95% confidence interval) in the children’s average moderate-to-vigorous physical activity minutes per day in Year 4 associated with parents’ moderate-to-vigorous physical activity in Year 4 and Year 1 for those with complete data. **Table S3.** Mean difference (95% confidence interval) in the children’s change in sedentary minutes per day between Year 1 and Year 4 associated with parents’ change in sedentary time between Year 1 and Year 4 for those with complete data. **Table S4.** Mean difference (95% confidence interval) in the children’s change in moderate-to-vigorous physical activity minutes per day between Year 1 and Year 4 associated with parents’ change in moderate-to-vigorous physical activity between Year 1 and Year 4 for those with complete data. (DOCX 25 kb)
