# Supplementary material for: Association of parents’ and children’s physical activity and sedentary time in Year 4 (8–9) and change between Year 1 (5–6) and Year 4: a longitudinal study
Source: Int J Behav Nutr Phys Act. 2017 Aug 17;14:110. doi: 10.1186/s12966-017-0565-0 (PMC5561613; doi:10.1186/s12966-017-0565-0)
Supplement: Additional file 1: — Table S1. Mean difference (95% confidence interval) in the children’s average sedentary minutes per day in Year 4 associated with parents’ sedentary time in Year 4 and Year 1 for those with complete data. Table S2. Mean difference (95% confidence interval) in the children’s average moderate-to-vigorous physical activity minutes per day in Year 4 associated with parents’ moderate-to-vigorous physical activity in Year 4 and Year 1 for those with complete data. Table S3. Mean difference (95% confidence interval) in the children’s change in sedentary minutes per day between Year 1 and Year 4 associated with parents’ change in sedentary time between Year 1 and Year 4 for those with complete data. Table S4. Mean difference (95% confidence interval) in the children’s change in moderate-to-vigorous physical activity minutes per day between Year 1 and Year 4 associated with parents’ change in moderate-to-vigorous physical activity between Year 1 and Year 4 for those with complete data. (DOCX 25 kb) [file 12966_2017_565_MOESM1_ESM.docx]

**Table S1 Mean difference (95% confidence interval) in the children's average sedentary minutes per day in Year 4 associated with parents’ sedentary time in Year 4 and Year 1 for those with complete data***

| **Exposure** | | **Child’s sedentary time in Year 4 (mins/day)** | | | |
| --- | --- | --- | --- | --- | --- |
|  |  | **All**  Mean difference (95% CI) | **Boys**  Mean difference (95% CI) | **Girls**  Mean difference (95% CI) | **P for gender interaction** |
| **Parent’s sedentary time in Year 4 (mins/day)** | |  |  |  |  |
| **Female parent** | | N=544 | N=246 | N=298 |  |
|  | Model 1 | 0.22 (-0.00, 0.43) | 0.20 (-0.13, 0.53) | 0.23 (-0.05, 0.50) | 0.91 |
|  | Model 2 | 0.21 (-0.00, 0.43) | 0.19 (-0.14, 0.52) | 0.23 (-0.03, 0.50) | 0.93 |
| **Male parent** | | N=295 | N=143 | N=152 |  |
|  | Model 1 | 0.19 (-0.07, 0.44) | 0.48 (0.11, 0.84) | 0.03 (-0.06, 0.13) | 0.02 |
|  | Model 2 | 0.20 (-0.08, 0.49) | 0.51 (0.14, 0.88) | 0.02 (-0.08, 0.11) | 0.02 |
| **Parent’s sedentary time in Year 1 (mins/day)** | |  |  |  |  |
| **Female parent** | | N=320 | N=148 | N=172 |  |
|  | Model 1 | 0.15 (0.02, 0.29) | 0.17 (-0.02, 0.36) | 0.14 (-0.04, 0.31) | 0.81 |
|  | Model 2 | 0.12 (-0.05, 0.29) | 0.12 (-0.15, 0.39) | 0.12 (-0.05, 0.28) | 0.93 |
| **Male parent** | | N=200 | N=87 | N=113 |  |
|  | Model 1 | -0.02 (-0.15, 0.12) | 0.09 (-0.06, 0.24) | -0.08 (-0.34, 0.17) | 0.34 |
|  | Model 2 | -0.03 (-0.20, 0.14) | 0.11 (-0.04, 0.25) | -0.14 (-0.48, 0.20) | 0.26 |

^*^ Model 1 is adjusted for child’s age at Year 4 and gender;

Model 2 is additionally adjusted for the child’s BMI z score, number of siblings, household IMD score and the female/male parent’s age and BMI at Year 4 for models with parent’s sedentary time in Year 4 as the exposure, or for child’s BMI z score, number of siblings, household IMD score and the female/male parent’s age and BMI at Year 1 for models with the parent’s sedentary time in Year 1 as the exposure

**Table S2 Mean difference (95% confidence interval) in the children's average moderate-to-vigorous physical activity minutes per day in Year 4 associated with parents’ moderate-to-vigorous physical activity in Year 4 and Year 1 for those with complete data^*^**

| **Exposure** | | **Child’s MVPA in Year 4 (mins/day)** | | | |
| --- | --- | --- | --- | --- | --- |
|  |  | **All**  Mean difference (95% CI) | **Boys**  Mean difference (95% CI) | **Girls**  Mean difference (95% CI) | **P for gender interaction** |
| **Parent’s MVPA in Year 4 (mins/day)** | |  |  |  |  |
| **Female parent** | | N=544 | N=246 | N=298 |  |
|  | Model 1 | 0.22 (0.13, 0.30) | 0.21 (0.07, 0.35) | 0.22 (0.12, 0.33) | 0.89 |
|  | Model 2 | 0.22 (0.13, 0.30) | 0.21 (0.08, 0.35) | 0.23 (0.12, 0.34) | 0.92 |
| **Male parent** | | N=295 | N=143 | N=152 |  |
|  | Model 1 | 0.23 (0.12, 0.33) | 0.18 (-0.03, 0.38) | 0.28 (0.16, 0.40) | 0.49 |
|  | Model 2 | 0.23 (0.12, 0.34) | 0.19 (0.01, 0.38) | 0.28 (0.14, 0.41) | 0.49 |
| **Parent’s MVPA in Year 1 (mins/day)** | |  |  |  |  |
| **Female parent** | | N=320 | N=148 | N=172 |  |
|  | Model 1 | 0.08 (-0.02, 0.17) | -0.08 (-0.27, 0.11) | 0.18 (0.07, 0.30) | 0.04 |
|  | Model 2 | 0.07 (-0.02, 0.17) | -0.07 (-0.27, 0.13) | 0.18 (0.06, 0.29) | 0.03 |
| **Male parent** | | N=200 | N=87 | N=113 |  |
|  | Model 1 | 0.11 (0.00, 0.22) | 0.06 (-0.24, 0.36) | 0.15 (0.04, 0.25) | 0.61 |
|  | Model 2 | 0.11 (-0.02, 0.23) | 0.08 (-0.25, 0.41) | 0.14 (0.04, 0.24) | 0.72 |

^*^ Model 1 is adjusted for child’s age at Year 4 and gender;

Model 2 is additionally adjusted for the child’s BMI z score, number of siblings, household IMD score and the female/male parent’s age and BMI at Year 4 for models with parent’s sedentary time in Year 4 as the exposure, or for child’s BMI z score, number of siblings, household IMD score and the female/male parent’s age and BMI at Year 1 for models with the parent’s sedentary time in Year 1 as the exposure

**Table S3 Mean difference (95% confidence interval) in the children's change in sedentary minutes per day between Year 1 and Year 4 associated with parents’ change in sedentary time between Year 1 and Year 4 for those with complete data***

| **Exposure** | | **Child’s change in sedentary time Year 1 to Year 4 (mins/day)** | | | |
| --- | --- | --- | --- | --- | --- |
|  |  | **All**  Mean difference (95% CI) | **Boys**  Mean difference (95% CI) | **Girls**  Mean difference (95% CI) | **P for gender interaction** |
| **Parent’s change in sedentary time Year 1 to Year 4 (mins/day)** | |  |  |  |  |
| **Female parent** | | N=222 | N=105 | N=117 |  |
|  | Model 1 | 0.09 (-0.06, 0.24) | 0.03 (-0.13, 0.19) | 0.14 (-0.05, 0.33) | 0.23 |
|  | Model 2 | 0.08 (-0.07, 0.23) | 0.07 (-0.16, 0.29) | 0.13 (-0.05, 0.32) | 0.47 |
| **Male parent** | | N=105 | N=46 | N=59 |  |
|  | Model 1 | 0.07 (-0.03, 0.17) | 0.18 (-0.36, 0.72) | 0.04 (-0.06, 0.14) | 0.61 |
|  | Model 2 | 0.06 (-0.05, 0.17) | 0.26 (-0.33, 0.84) | 0.02 (-0.07, 0.11) | 0.60 |

^*^ Model 1 is adjusted for child’s age at Year 1 and gender;

Model 2 is additionally adjusted for the child’s BMI z score, number of siblings, household IMD score and the female/male parent’s age and BMI at Year 1

| **Exposure** | | **Child’s change in MVPA Year 1 to Year 4 (mins/day)** | | | |
| --- | --- | --- | --- | --- | --- |
|  |  | **All**  Mean difference (95% CI) | **Boys**  Mean difference (95% CI) | **Girls**  Mean difference (95% CI) | **P for gender interaction** |
| **Parent’s change in MVPA Year 1 to Year 4 (mins/day)** | |  |  |  |  |
| **Female parent** | | N=222 | N=105 | N=117 |  |
|  | Model 1 | 0.15 (0.03, 0.27) | 0.23 (-0.03, 0.48) | 0.09 (-0.05, 0.23) | 0.38 |
|  | Model 2 | 0.14 (0.03, 0.26) | 0.22 (-0.04, 0.48) | 0.08 (-0.06, 0.22) | 0.37 |
| **Male parent** | | N=105 | N=46 | N=59 |  |
|  | Model 1 | 0.05 (-0.10, 0.21) | 0.12 (-0.11, 0.36) | -0.04 (-0.22, 0.14) | 0.24 |
|  | Model 2 | 0.05 (-0.11, 0.20) | 0.13 (-0.08, 0.34) | -0.07 (-0.26, 0.13) | 0.22 |

**Table S4 Mean difference (95% confidence interval) in the children's change in moderate-to-vigorous physical activity minutes per day between Year 1 and Year 4 associated with parents’ change in moderate-to-vigorous physical activity between Year 1 and Year 4 for those with complete data^*^**

^*^ Model 1 is adjusted for child’s age at Year 1 and gender;

Model 2 is additionally adjusted for the child’s BMI z score, number of siblings, household IMD score and the female/male parent’s age and BMI at Year 1
